# Valorization of mixed blackwater/agricultural wastes for bioelectricity and biohydrogen production: A microbial treatment pathway

**DOI:** 10.1016/j.heliyon.2024.e41126

**Published:** 2024-12-12

**Authors:** G. Plason Z. Plakar, Abdulsalami S. Kovo, Kanayo L. Oguzie, Emeka E. Oguzie

**Affiliations:** a African Centre of Excellence in Future Energies and Electrochemical Systems (ACE-FUELS), Federal University of Technology, Owerri, PMB 1526, Imo State, Nigeria; b Federal University of Technology Owerri, Imo State, Nigeria; c Department of Chemistry, Emmet A. Dennis College of Natural Sciences, Cuttington University, Gbarnga City, Liberia; d Federal University of Technology Minna, Minna, Nigeria

**Keywords:** Blackwater/Agricultural Waste, Microbial treatment, Microbial fuel cell, Biohydrogen, Bioelectricity

## Abstract

The management of wastewater and agricultural wastes has been limited by the separate treatment processes, which exacerbate pollution and contribute to climate change through greenhouse gas emissions. Given the energy demands and financial burdens of traditional treatment facilities, there is a pressing need for technologies that can concurrently treat solid waste and generate energy. This study aimed to evaluate the feasibility of producing bioelectricity and biohydrogen through the microbial treatment of blackwater and agricultural waste using a dual-chamber Microbial Fuel Cell (MFC). The research focused on identifying optimal feedstock ratios and pH conditions, accompanied by biochemical assays to characterize the microbial community involved. The predominant microorganisms identified included *Escherichia coli*, *Salmonella* spp., and *Pseudomonas aeruginosa*, among others. The highest open circuit voltage achieved was 1090 mV at a hydraulic retention time (HRT) of 6 days. Maximum removal efficiencies for Chemical Oxygen Demand (COD) and Biochemical Oxygen Demand (BOD) were 90.87 % and 76.67 %, respectively, with a Columbic efficiency of 40.17 %. The peak power density measured was 345 mW/m^2^, and the highest hydrogen yield was 483 ppm/s. The optimal feedstock ratio was found to be 3:1:1 (300 g cassava peel, 100 g banana peel, and 100 g tomato waste), with ideal pH conditions at 9.35. This study underscores the potential for generating bioelectricity and biohydrogen from the microbial treatment of mixed blackwater and agricultural wastes in a single system, eliminating the need for separate treatment and the use of external energy source. The work contributes to the advancement of environmental engineering and management, bioenergy, microbial fuel cell, and affordable and clean energy.

## Graphical abstract

**Figure:**
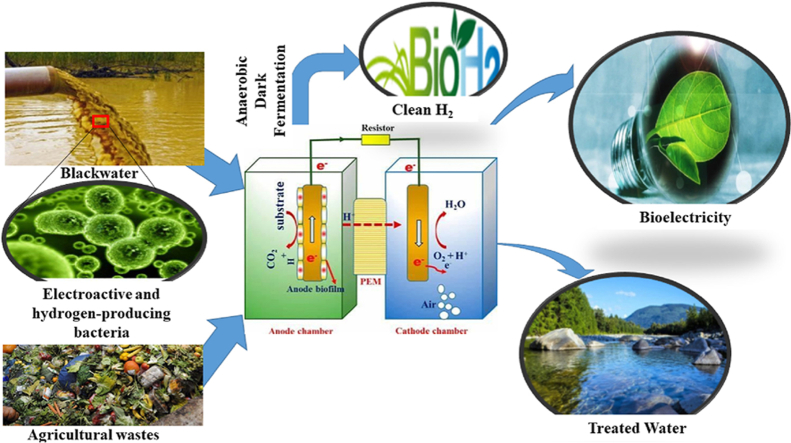

## 1 Introduction

The global capacity to manage waste efficiently is continuously strained by its growing population [1,2]. Agricultural waste streams and municipal wastewater, particularly blackwater, are two significant causes of this problem [3]. Globally, an estimated 931 million tons of food (17 %) are wasted yearly, with 61 % coming from homes, 26 % from food service, and 13 % from retail [4]. Similarly, $359.4\times 10^{9}m^{3}$ of wastewater is produced annually from which only $52\%\;(188.1\times 10^{9}m^{3}/yr$) is treated while over $48\%\;\left(171.3\times 10^{9}m^{3}/yr\;\right)$ is released to the environment untreated [5]. Blackwater, mostly made up of household sewage and agricultural wastes carries substantial organic load, and bacteria, and emits greenhouse gases, which can harm the environment and human health if not adequately treated.

Fecal virus contamination of surface waterways is just one aspect of the pollution issue. For instance, Hellmér et al. [6] reported fecal virus infections ranging from asymptomatic, mild forms to severe gastroenteritis, conjunctivitis, hepatitis, respiratory disease, and central nervous system infections. Worth noting are the notorious human adenoviruses (HAdV), which make up 88 different genotypes [7] and have been linked to pneumonia, respiratory tract infections, conjunctivitis, and gastroenteritis [8]. Not surprisingly, Rotavirus (RV) has been reported as the most important cause of severe diarrhea among young children and infants [9] with at least 500,000 deaths in 2004 according to a WHO report predominantly occurring in developing countries.

In addition, Wear et al. [10] found that sewage discharge contains a variety of other contaminants, including heavy metals, pathogens, endocrine disruptors, and medications, which can pollute the environment. Meador et al. [11] found that at least 31.25 % of contaminants from sewage treatment plants were detected in nearby waters in Puget Sound, Washington U.S.A. Similarly, poorly managed agricultural wastes can pollute air and water with excess nutrients and greenhouse gases. Investigations into the potential causes of birth defects and respiratory conditions like asthma have focused on these landfill sites [12]. Incinerators have also been connected to these ailments while bronchitis and other lung-related illnesses have been linked to environmentally illegal composting and material recycling facilities due to odors [12].

Although these wastes pose serious environmental and public health concerns, they also show significant promise for clean energy production. According to Logan et al. [13,14] domestic wastewater, including blackwater, is capable of generating clean energy up to 2 kW/h.m^3^. Therefore, instead of merely treating blackwater/agricultural wastes, there is a need to harness the energy potential of these waste streams using cost-effective methods [15,16].

Various methods are employed to transform these wastes into energy products [[12], [13], [14]]. Among them, Microbial treatment particularly anaerobic digestion powered by Microbial Fuel Cells, has received extensive attention due to its potential to convert waste biomass to energy without emitting any environmental pollutants [17,[18], [19], [20]]. Additionally, anaerobic dark fermentation has been employed to produce clean biohydrogen (BioH_2_) [18,19]. When integrated, both microbial treatment and anaerobic dark fermentation are used to treat both solid and liquid wastes using Microbial Fuel Cell (MFC) technology [21]. MFC can produce bioelectricity and biohydrogen while treating wastes as it involves the biological breakdown of agricultural wastes into simple sugars, later converted to electricity and protons [22]. On the other hand, Microbial Electrolysis Cells (MECs) can be used for producing biohydrogen by simply applying external volt (0.4–1.11 V) [23], attracting new research due to their versatilities in co-treating other waste streams, henceforth called bioremediation [22]. While this technology remains at the heart of current research, the biohydrogen production pathway is attractive due to its prime independence of external electricity supply. BioH_2_ has been regarded as the fuel of the future as it is clean and renewable and possesses a relatively high-energy content per unit weight (122–142 kJ/g) than conventional fossil fuels [24,25].

One common method for producing BioH_2_ involve the integration of MFC with MEC, where the electricity produced from the MFC is used for supplying the MEC with electricity for BioH_2_ production, utilizing either solid or liquid waste streams [26]. Even so, some progress has been made in integrating bioelectricity and biohydrogen in a single continuous MFC. Recently, Guadarrma-Perez et al. [27] evaluated the production of bioelectricity and biohydrogen from xylose using both single culture and mixed culture and found that the system could produce a maximum voltage of 671 mV at 1000 Ω resistance and power density and biohydrogen rate of 67 mW/m^2^ and 5.2L H_2_/L.d (or 2.39 mol H_2_/mol of sucrose), respectively.

Nevertheless, there is a dearth of data available on the potential of co-treating both solid agricultural wastes and liquid waste (blackwater) to coproduce bioelectricity and biohydrogen in a dual-chamber microbial fuel cell. Other works have highlighted the biohydrogen and bioelectricity production from several single-phase waste streams such as soil, wastewater, compost, and anaerobic sludge using mixed culture; and xylose using both pure and mixed culture [28,29]. In this study, a mixed culture (electrigens and HPBs) sourced from the blackwater samples was used. The purpose of this study was to assess the possibility of producing bioelectricity and biohydrogen from the microbial co-treatment of blackwater and agricultural wastes using a dual-chamber MFC and to determine the optimum feedstock ratio and pH conditions.

## 2 Materials and methods

3 L of Blackwater was collected from the FUTO Guest House Septic tank in Imo state, Nigeria, homogenized, and stored in PET bottles (maximum 1.5 L). Agricultural wastes consisting of banana peels, cassava peels, and tomato residues were collected from a local market, blended at 5000 rpm, homogenized for 5 min, and transferred to the anodic chamber. The compositions of each setup for the samples are shown in Table S1. The final mixture was fully homogenized and subjected to N_2_ purging to achieve anaerobic conditions (room temperature: 26±3 °C).

All chemicals and reagents used in this work were analytical grades purchased from Sigma Aldrich and Thermo Scientific, and all purities were chosen based on the method and intended use without further purification.

### 2.1 Microbial characterization

Differential and structural stains and observation of colonial/cultural characteristics by cultivating on enriched, differential, and selective media are usually employed for genus-level identification of bacterium and yeast. Biochemical tests are required for a species-level determination as they can detect enzymes because different species produce fingerprint enzymes unique to that organism alone [30]. These tests can even detect organisms that catalyze complex carbohydrates, sugars, lipids, proteins, and other organic matter. Biochemical tests conducted included Durham Tube Sugar Fermentation Test, Methyl Red Test, Voges-Proskauer Test, Catalyst Test, Oxidase Test, Nitrate Reduction Test, Oxidation-Fermentation Test, Starch Hydrolysis Test, Urea Hydrolysis, Indole Test (Tryptophan Hydrolysis Test), DNase Test (DNA Hydrolysis Test), Hydrogen Sulfide Production Test (H_2_S), Citrate Utilization Test, Decarboxylase Test [[31], [32], [33]]. All tests were co-performed with a control (media and blank) to account for contamination, dehydration, and deterioration.

A Durham Tube was used for the sugar fermentation test with Phenol red as an indicator. First, an isolate was inoculated into a test tube containing phenol indicator and then incubated at 35 °C for 24 h as detailed elsewhere in the literature [34]. The carbohydrate source was mixed wastewater and agricultural wastes. A color change of the Phenol indicator from red (at neutral pH) to yellow (at acidic pH and) and subsequent gas production within the Durham tube was noted.

Methyl Red (MR) and Voges-Proskauer (VP) Tests were carried out to identify organisms in the *E. coli* group (MR positive) and those in the Enterobacter- Klebsiella group (MR negative) as detailed elsewhere [32]. First, a 0.02 % MR indicator was prepared, after which the MR-VP broth was brought to room temperature. Two separate tubes of MR-VP broths were inoculated and incubated at 35°C for 48 h followed by an addition of methyl red indicator to tube one whereas Barritt's reagent was used for tube two for the Voges-Proskauer (VP) test. Caution was taken to avoid over-inoculating because it increases the number of viable cells, and incubating for insufficient time may result in a false-positive MR test. In some rare cases, certain organisms, such as *Hafnia alvei* and *Proteus mirabilis*, may show both a positive MR and positive VP reaction, which is often delayed [35]. The color changes were noted.

The catalyst test (CT) is used to determine if an organism is capable of breaking down hydrogen peroxide (H2O2) into water (H2O) and oxygen (O2). A catalyst (+) organism will decompose H2O2 into hydrogen and oxygen, while a catalyst (−) will not show any bubbles, indicating its inability to decompose hydrogen peroxide. Catalase-positive organisms are either aerobes or facultative, which use the enzyme catalase to neutralize the effects of toxic bactericides such as hydrogen peroxide as a protection against oxidative damage. 1 ml of 3 % H2O2 was added to a test tube. Using the inoculating loop, a small amount of organisms from a well-isolated 18 to 24-h colony was collected and placed into the test tube. The tube was placed against a dark background and observed for immediate O2 bubble formation or effervescence at the end of the inoculating loop. If a bubble is formed, it indicates catalase positive. Since anaerobes lack this enzyme, this test can confirm if anaerobes or facultative anaerobes are present (catalase negative) or aerobes and facultative bacteria are present (catalase positive) [36].

Oxidase is an intracellular substance that transfers electrons to oxygen to form water. Oxidase activity can be found in aerobic, facultative, anaerobic, and microaerophilic bacteria. The test identifies *Pseudomonas, Neisseria, Vibrio, Alcaligenes, campylobacter, Pasteurella, Flavobacterium, and Aeromonas*, which are all oxidase-positive, while the members of *Enterobacteriaceae* are exclusively negative for this character. The entire plate method was used in this work [31]. 2–3 drops of pinkish oxidase test reagent (electron acceptor) were added to bacteria agar plates of *P. aeruginosa* and *E. coli* such that the reagent covered the colonies of both organisms. The color change was observed within 10–30 s. A pink-to-bluish color change indicates the presence of cytochrome activity for a positive test. In *Pseudomonas aeruginosa*, the colonies first become pink, then moron, dark red, and then dark purple or black, revealing it has the enzyme oxidase (or it is oxidase positive). *E.coli* shows unchanged colors, indicating it is oxidase negative [31].

Nitrate-reducing bacteria convert nitrate (NO_3_⁻) to nitrite (NO_2_⁻) during metabolic processes to obtain nitrogen, using nitrate as a hydrogen acceptor with the enzyme nitrate reductase. This is important for identifying bacteria like *Neisseria, Haemophilus, Shewanella putrefaciens*, and *Branhamella*, as well as denitrifying bacteria like *Pseudomonas.* Nitrate broth was inoculated with the target organism and incubated for 24–48 h at 35 °C. Adding α-naphthylamine and sulfanilic acid shows a red color if nitrite is present. The denitrification test checks for nitrogen gas in a Durham tube, where bubbles indicate a positive result. Zinc dust can also be added: if the broth turns red, it means nitrate is still present; if there's no color change, nitrate has been fully reduced, confirming successful denitrification [31].

The O-F test is used to determine the aerobic or anaerobic nature of a microbe, a feature used in the identification of bacteria and yeasts. It distinguishes Gram-negative rods (aerobes) such as *P. aeruginosa* from those that are facultative such as *E. coli*, Gram-positive cocci such as *Micrococcus* spp. (*aerobes*) from those of *staphylococcus spp*. that are facultative anaerobes. A sterile needle was used to inoculate Hugh and Leifson O-F tube medium (2 tubes/organisms) and then one tube was covered with mineral oil and incubated at 35 °C for 48 h for a color change from green to yellow. A yellow color indicates a facultative/anaerobic organism degrading glucose via a facultative pathway. If a yellow color appears in the uncovered tube, it indicates the bacterium is oxidative [31]. If both tubes (covered and uncovered) remain green, it signals an asaccharolytic (inert) organism.

The Starch Hydrolysis Test (SHT) is used to test the breakdown of starch by amylase. Starch agar medium was melted, cooled to 45 °C, and poured into sterile dishes. The medium was allowed to solidify before labeling with the desired organism. Using a sterile technique, a single streak inoculation was done for each organism into the center of its appropriately labeled plates. The inoculated bacteria was incubated at 37 °C for 48 h and the fungal inoculated plate at 25 °C for 72–96 h [31]. The surface of the plates was flooded with Gram's iodine using a dropper. The plates were immediately examined after adding the Gram's iodine for any color change along the growth line of the microorganism. If starch is hydrolyzed by the amylase produced by microorganisms, a clear zone will be produced around the growth, indicating the presence of starch-digesting enzymes. A dark blue coloration, for example by *E. coli*, indicates a negative reaction.

When a hydrolytic enzyme called urease breaks down urea, alkaline products including ammonium and carbon dioxide are formed. It is used to differentiate fungi from different groups of bacteria since a 24-h urea breakdown is a trait used to distinguish *proteus spp*. from other enteric bacteria. Usually, urea broth containing yeast extract, urea, and phenol red indicator is inoculated and then a color change is observed from yellow-orange to pinkish-red after 24h of incubation at 37 °C. Here, two tubes of urea agar medium/agar broth were each labeled with the desired inoculating bacteria name. The inoculation was done on one urea agar slant/broth with *proteus vulgaris* and the other with *E. coli*; incubated at 37 °C for 24 h. The slants/broth were examined for color change of the medium. A dark pink color shows a positive reaction while a no color change is classified as a negative test. *Proteus* shows positive color change while *E. coli* show no color change [32].

The indole test is used to characterize enteric bacteria. Tryptophan is oxidized by the enzyme tryptophans to indole, pyruvic acid, and ammonia. It is used to differentiate the indole-positive enteric (*E. coli and P. vulgaris*) from the indole-negative enteric (*Enterobacter aerogenes* and others). An isolate is inoculated on a SIM medium tube and the production of indole (red color) is observed with Kovac's reagent. Two SIM broth tubes were inoculated with *E. coli* and *E. aerogenes*, while the third was kept as uninoculated control. These were incubated at 35 °C for 24–48h before adding 1 ml of Kovac's reagent to each tube. The tubes were shaken gently, allowed to stand so that the reagent would flow, and was observed for red-violet ring interface just between the reagent maxima and the broth and the reagent. A positive indole test is shown by the presence of a red-violet ring adding the reagent. A yellow color or no color represents a negative test [31].

In the DNase test, several inoculum of the microorganisms is used to inoculate an agar plate that contains DNA and the metachromatic dye toluidine blue O. After a 24-h incubation, a positive test can be detected by the formation of a pink halo around the colony. A heavy suspension of the organism is spotted on the left side of the DNA agar plate followed by inoculation on the other side of the DNA test agar plate [33]. The inoculated agar plates were incubated at 35 °C for 24 h and were observed for growth or color change. A pink halo around the growth of the bacterium is the result of the bacterium's growth due to the hydrolytic action of the DNase on the DNA while no color change indicates a negative reaction.

The Hydrogen Sulfide (H2S) test is used to differentiate *S. Typhimurium* and *P. vulgaris* (H2S producers) from *E. coli* and *Shigella flexneri* as well as another member of *Enterobacteriaceae*. It is performed by inoculating a culture medium with Fe as an indicator for H2S on amino acid cysteine as the sulfur substrate incubated at 35 °C for 24–48 h and observing the medium for blackish region or black precipitates [33]. The blackening of the tube indicates H2S (+) ve while no blackening indicates H2S negative.

The citrate test uses the presence of citrase enzyme to differentiate intestinal bacteria such as *S. typhimurium and E. aerogenes*, which feed on citrate as a carbon source. The organism is inoculated into a citrate agar plate or slant, incubated at 35 °C for 24–48 h, subjected to bromothymol blue indicator, and observed for color change from green to blue. Citrate-positive organisms display growth and a blue color after 24–48 h [31]. *E. coli* shows no color change while E. aerogenes show a color change to blue.

Glucose undergoes fermentation to produce acids due to anaerobic bacteria activity, reducing the pH (yellow color reaction), and a condition that favors decarboxylase formation. Decarboxylases act as amino acids (lysine) to produce alkaline amines as their final product, which increases the pH and changes bromothymol purple from yellow to purple (carboxylase positive). A purple color indicates a positive test and alkaline condition whereas a yellow color (acidic condition) indicates there are no carboxylases [31,33].

### 2.2 MFC setup and operation

**Fig. 1:**
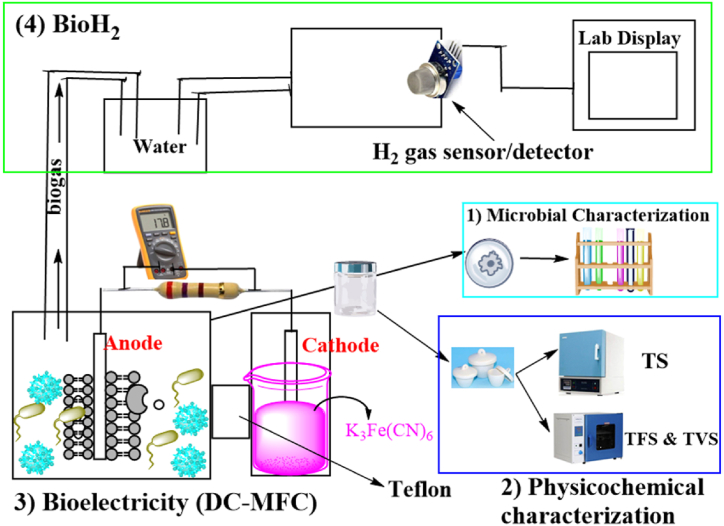
Experimental Flowchart. Microbial experiment characterization (1), physicochemical characterization (2), bioelectricity generation (3), and biohydrogen rate measurement (4). Fig. 1 was not drawn to scale.

An H-shape, mediator-free dual-chamber microbial fuel cell made of pixel glass was used for microbial treatment, bioelectricity generation, and biohydrogen production. A pair of cylindrical glasses (500 mL each) formed the anodic and cathodic chambers of MFC-1 with a stainless steel-metallic clamp to hold the joint of the two chambers containing the Teflon membrane in place. Three pairs of PET boxes (1000 mL each) were used to construct MFC-2, MFC-3, and MFC-4 with PVC-20s fitted to the two chambers to hold the Teflon (AFT Fluorotec, United Kingdom) membrane in place. Fig. 1 shows the experimental setup for this study.

Graphite sheet with dimensions 5×4×0.5cm was used as the anode due to its suitability for electron transfer, thermal and chemical stability, and cost-effectiveness [37], while a carbon cloth (CMI-7000, Membrane International Inc. USA) with dimensions 5×4×0.5cm was used as the cathode material as it has a large surface area, has high electrical conductivity, is corrosion resistant, and cheap [38]. The anode-cathode distance was 3 cm, consistent with the 1–5 cm range suggested by Chen et al. [39] and Logan and Ann [40] since lower anode-cathode distances have proven to increase power production [39]. Potassium ferrocyanide K_3_Fe(CN)_6_, 50 mM [41], was used as an electron acceptor in the cathode due to its high performance. Compared to Pt-based catalysts, K_3_Fe(CN)_6_ is cheap and readily accessible. The proton exchange membrane used was the Teflon membrane already pretreated according to Chen et al. [42] due to its high mechanical resistance, suitable operational flexibility at elevated temperatures, and cheapness. A DT90205A Digital Multimeter was connected to both ends of the anode and cathode wires (R = 4.0 Ω). The digital multimeter (DT9205A) was capable of measuring up to 1000 V, 10-A current, and 250 Ω resistance in the DC-MFC and displaying the results in the form of trends. It is preferred to analog multimeters due to its high accuracy and versatility. All MFCs were operated at 27 ±3 ^o^C. The rate of anodic gas or hydrogen gas production was monitored continuously using the MQ-8 H_2_ gas sensor.

### 2.3 Analytical methods

Characterization of the combined feedstock was done as described in the Standards Methods for the Examination of Water and Wastewater [43]. In short, samples collected were either used as received or diluted as with BOD calculation.

The microbial consortia in the blackwater were characterized using biochemical tests as described elsewhere in the literature [44]. Altogether, samples were diluted using a dilution factor of 10^6^ and then subjected to biochemical tests to determine the genus of microbes inhabiting the blackwater samples.

Real-time measurements of the open circuit voltage produced from each MFC setup were obtained from the DT9025A digital multimeter and recorded every 3 h for eight days. The average open circuit voltage was calculated and tabulated. The current *I* (mA) was measured using. $I=V/R_{ex}$ [45], where V (mV) is the voltage and Rex is the external resistance. The areal power density P (mW/m^2^) and areal current density *Ϳ* (mA/m^2^) were calculated according to $P=\frac{I\times V}{A}\times 1000$ [45] and $J=\frac{V\times A}{R_{ex}}$ [45], where A (m^2^) is the surface area of the anode. The Columbic efficiency was calculated according to $\text{CE}=\frac{M\int_{0}^{t}I\text{dt}}{\text{Fb}V_{\text{An}}\Delta \text{COD}}$ [46], where M is the molecular weight of oxygen and M=32, F is Faraday's constant, b is the number of electrons exchanged per/mole of oxygen and b=4, V_An_ is the volume of liquid in the anode compartment, and ΔCOD is the change in COD over time t_b_. The percentage removal of COD and BOD were each determined according to (1), (2), respectively [43].

(1) $$\%\text{COD}\;\text{removal}=\frac{{\text{COD}}_{\mathrm{int}}-{\text{COD}}_{\text{final}}}{{\text{COD}}_{\mathrm{int}}}\times 100\%$$

(2) $$\%\text{BOD}\;\text{removal}=\frac{{\text{BOD}}_{\mathrm{int}}-{\text{BOD}}_{\text{final}}}{{\text{BOD}}_{\mathrm{int}}}\times 100\%$$

The H_2_ gas was collected and measured with an MQ-8 hydrogen sensor, an Arduino Leonardo board, and a computer. Additional calibration of the MQ-8 H_2_ sensor was not carried out at ambient conditions for two reasons. First, the deviations arising from changes in temperature and relative humidity at ambient conditions, when compared to the standard chromatographic technique as reported by Barrera et al. [47] was 14.44 ppm and error of 0.6 and 1 % [[48], [49], [50]] were not large enough to invalidate the results, which are more qualitative than quantitative. Second, although calibration would increase the precision and accuracy of the results, it is worth noting that the primary purpose of this work is to evaluate the potential of bioH_2_ production from mixed feedstock irrespective of the consistent deviations across all treatment conditions of the biohydrogen production rates. The results are reported in parts per million H_2_.

## 3 Results and discussions

### 3.1 Microbial characterization

Specific microorganisms exhibit distinct contributions during microbial treatment, which is imperative to have a full registry of all species present. Table 1 summarizes all species identified in the blackwater/agricultural waste sample.

**Table 1:** Summary of identified species from blackwater (BW)/Agricultural wastes (AW).

Sample	Microbial Isolate	Population (cfu/ml)
BW	*Pseudomonas aeruginosa*	0
BW + AW	*Pseudomonas aeruginosa*	55.0 x 10^4^
BW	*Escherichia coli*	3.3 x 10^3^
BW + AW	*Escherichia coli*	6.8 x 10^3^
BW	*Shigella* sp.	14.3 x 10^3^
BW + AW	*Shigella* sp.	54.6 x 10^5^
BW	*Enterobacter asburiae*	0
BW + AW	*Enterobacter asburiae*	66.4 x 10^5^
BW	*Klebsiella pneumoniae*	11.6 x 10^3^
BW + AW	*Klebsiella pneumoniae*	31.5 x 10^3^
BW	*Candida guillermondii*	59.7 x 10^4^
BW + AW	*Candida guillermondii*	57.4 x 10^4^
BW	*Pythium ultimum*	0
BW + AW	*Pythium ultimum*	29.6 x 10^4^

From Table 1, there was an increment in the total count of the microorganisms after the addition of the agricultural wastes, which probably resulted from an increase in the nutrient concentration under the same environmental conditions [51] with one exception to *Candida guillermondii*. *Candida guillermondii* feasts on n-alkanes, surviving at 30°C in aerobic conditions [52] and since the anodic chamber of the MFC was operated in anaerobic condition, the survivability of the yeast was hindered, causing its decrease. Its decrease is also attributed to the shortage of those nutrients (primarily n-alkanes) required for the growth of the yeast, leading to their reduction in the stationary phase [53]. The sudden appearance of *Pythium ultimum* is because these soil-borne plant pathogens usually rely on plants to meet their nutrient requirements; hence, they may remain on agricultural wastes and grow as fast as 1 mm/h [54] when introduced to a suitable environment (25−30°C).

The presence of these microbes in blackwater and their ability to decompose these wastes has sparked curiosity in their potential to drive bioelectricity and biohydrogen production in recent years. For instance, *Pseudomonas aeruginosa* is increasingly recognized for its role in bioelectricity production through phenazine redox mediators, even though the efficiency of the current generation varies with strain and substrate, with certain isolates showing superior capabilities [55].

*Escherichia coli* and *Enterobacter asburiae* have been proven to play a significant role in biohydrogen production while *Candida guilliermondii* is known for its role in biofuel production. *Escherichia coli* can produce hydrogen via anaerobic mixed-acid fermentation, with genetic modifications such as hydrogenase deletions enhancing this process [56]. On one hand, *Enterobacter asburiae* strains like *SNU-1* can produce high volumes of hydrogen and efficiently convert formic acid [57,58]. Electrochemically active biofilms formed by this microorganism can enhance bioelectricity generation [59]. On the other hand, *Candida guilliermondii*, or *Meyerozyma guilliermondii*, is a versatile yeast with biotechnological potential, capable of utilizing diverse carbon sources for biofuel production [60]. Known for its versatility, *Klebsiella pneumoniae* is noteworthy for its capability to produce bioelectricity and biohydrogen from various waste sources. *K. pneumoniae-FA2*, for example, demonstrated high hydrogen production in microbial fuel cells using food industry wastewater [61]. Another study indicated significant hydrogen yields from glycerol, highlighting its adaptability and versatility in bioenergy applications [62]. The remaining community members including *Shigella* spp. and *Pythium ultimum* are yet to be linked with either bioelectricity production, biohydrogen production or both**.**

Therefore, the community analysis reveals the potential of blackwater as an inoculum source in bioelectricity generation [63], wastewater treatment [64], and BioH_2_ generation via dark fermentation [44].

### 3.2 Microbial treatment efficiency

**Fig. 2:**
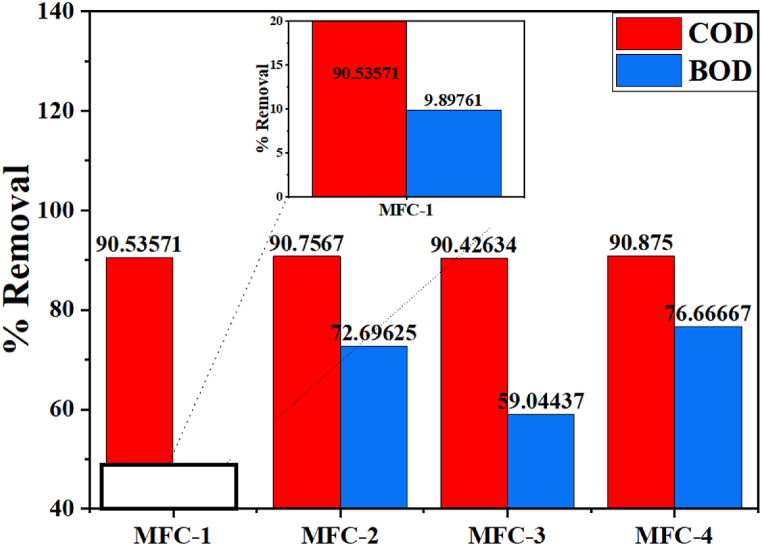
COD and BOD removal efficiency (in percentage) for MFC-1, MFC-2, MFC-3, and MFC-4 from blackwater/agricultural wastes over 8 days.

A reduction in the COD and BOD levels reflect the performance of MFC in degrading organic matter catalyzed by electroactive and fermentative bacteria and are presented as the % removal of COD and BOD. Fig. 2 shows the COD and BOD removal.

Fig. 2 shows the COD and BOD removal levels across four microbial fuel cells. Each MFC has different ratios of feedstock written as CP:TW:BP, where CP is cassava peel, TW is tomato wastes, and BP is banana peel (in multiple of 100). The ratio of each microbial fuel cell setup is given as MFC-1(1:1:1), MFC-2 (2:2:2), MFC-3 (2:1:3), and MFC-4 (3:1:1). The highest %COD and %BOD removals were observed from MFC-4 (90.87 % and 76.67 %), respectively. The lowest was observed from MFC-1 with 90.54 and 9.897 % COD and BOD, respectively. The removal of COD and BOD depends on the organic matter concentration correlated with the agricultural wastes and microbial community since other factors such as anode surface, mediators, membrane type, and environmental conditions like pH were constant across all four systems [48,49]. Cassava peel is rich in carbohydrates and more biodegradable compared to banana peel, which is superior to tomato waste, indicating that the overall carbohydrate content in MFC-4 was higher than MFC-2 [65]. The low BOD value observed in MFC-1 could be due to the limited quantity of blackwater, providing low moisture and motility for microbes and less biodegradable feedstocks [66]. Even though the difference in the percent removal for COD across all MFCs is not statistically significant (p >0.05), a realistic difference can be noted for the BOD results (p =0.01). The result agrees with Khan et al. [56] who reported COD removal in the range of 70−90% using KMnO_4_ and Anuforo et al. [57] reported a range of 73–76 % for BOD removal using C-C, C-Cu, and Cu-Cu electrodes in a double-chamber microbial fuel cell to treat piggery wastewater.

**Fig. 3:**
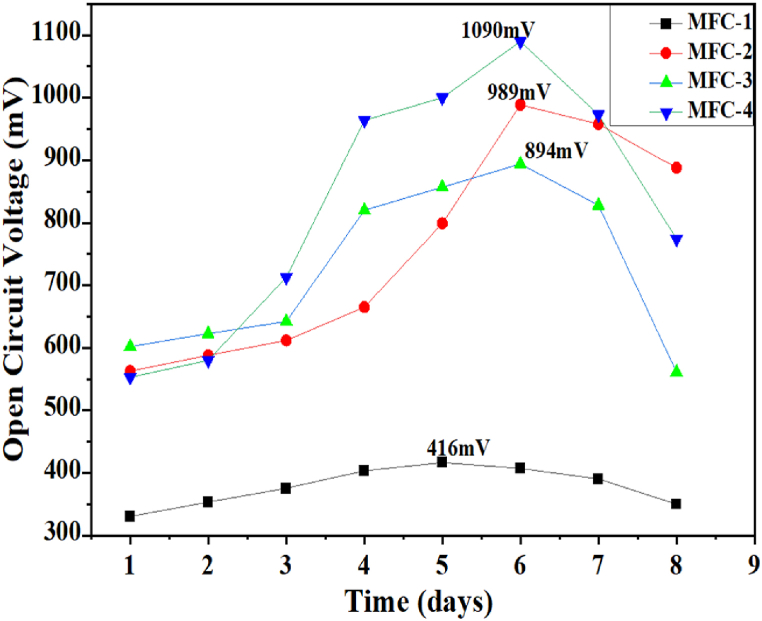
Open Circuit Voltage generated from all four setups of MFC (1, 2, 3, and 4) from t = 0–8 days.

The voltage generated by MFCs is a function of the conversion of chemical energy to electrical energy by electroactive bacteria [18]. A high open circuit voltage indicates the maximum voltage that can be harvested from the biocatalytic conversion of chemical energy to electrical energy when no load is attached. The open circuit voltage was generated within eight (8) days for all four setups (MFC-1, MFC-2, MFC-3, and MFC-4). A detailed description of the composition of each setup is provided in Table S1. The highest peak indicates the day on which the highest voltage was obtained as presented in Fig. 3.

Fig. 3 illustrates the open circuit potential recorded from 300 to 1100 mV for four miniaturized MFCs across varying hydraulic retention times (0–8 days). The maximum open circuit voltage (OCV) for MFC-3, MFC-2, and MFC-4 was observed at an HRT of 6 days, reaching 747 mV, 893 mV, and 1090 mV, respectively. In contrast, MFC-1 exhibited its maximum OCV of 416 mV on the 5th day. Notably, the highest OCV recorded was 1.09±0.184V from MFC-4. The average OCV values for the MFCs were 366.593 mV (MFC-1), 747.259 mV (MFC-2), 695.537 mV (MFC-3), and 816.482 mV (MFC-4). Consequently, the OCVs increase in the following order: MFC-1 < MFC-3 < MFC-2 < MFC-4. Statistically, the differences in open circuit voltage among the various MFCs are significant, indicating a considerable impact of substrate ratio on the voltage produced. A similar OCV value was reported by Pan et al. [67], where the lysinibacillus *xylanilyticus strain nbpp1* generated 1127 mV, achieving a maximum power density of 6.71 mW/cm^2^ at a current density of 11.14 mA/cm^2^. Thus, our findings align with the existing literature, presenting a competitive option for utilizing combined blackwater and agricultural wastes.

**Fig. 4:**
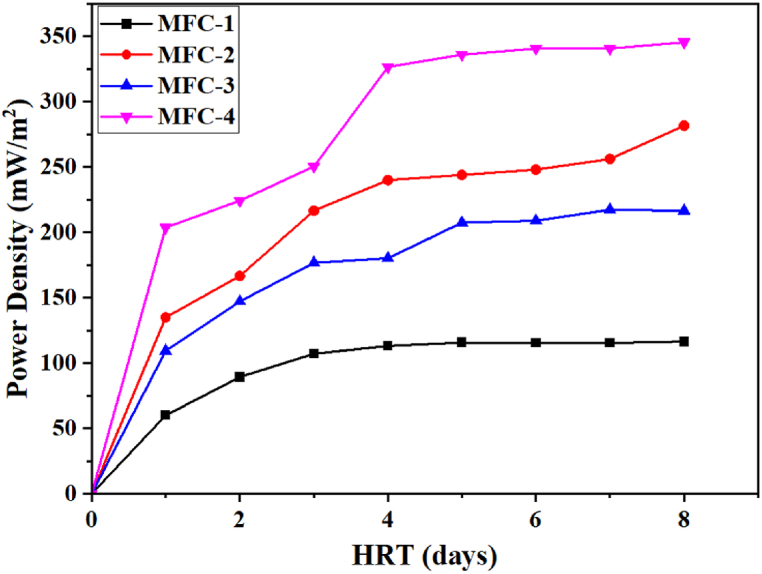
Power density across a 100-Ω resistor from t_i_ = 0 to t_f_ = 8 days for all four MFC (1, 2, 3, and 4) setups.

The areal power density (Dp, mW/m^2^) of the cell was calculated according to the previously described mathematical equation [68] using the cross-sectional area of the anode material. A high power density indicates a system is capable of yielding adequate amounts of energy based on the cross-sectional area of the anode while a lower value underscores the limitation of the microbial fuel cell. The power density for all MFCs is shown in Fig. 4.

From Fig. 4, MFC-4 produced the maximum power density (345 mW/m^2^) and MFC-1 (116.48 mW/m^2^) produced the least. When examining the influence of various chemical oxygen demands on microbial fuel cells using leachate as a substrate, Ishaq et al. [57] made a similar observation on the power density (339.41 mW/m^2^); however, the power density reported in this work is superior.

**Fig. 5:**
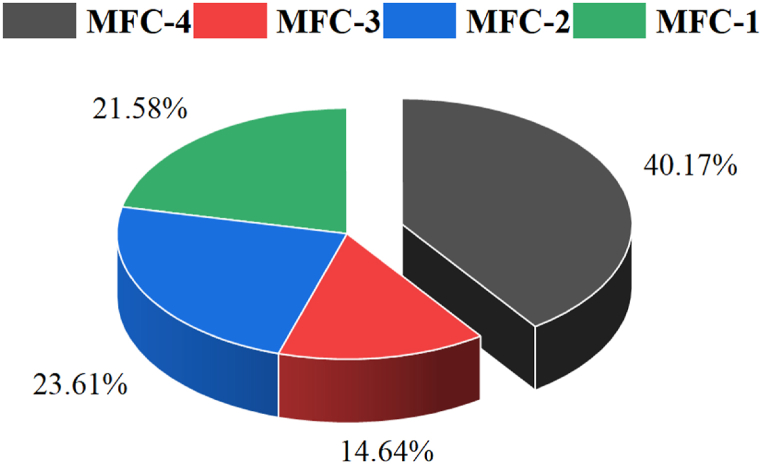
Columbic Efficiency, CE, at an external resistance (R_ext_ = 100 Ω) for all four MFCs.

The Columbic efficiency (CE) is useful for assessing the performance of a microbial fuel cell in converting chemical energy to electrical energy. A higher Columbic efficiency indicates that the MFC is efficiently converting organic matter into electricity while a lower Columbic efficiency indicates loss of energy or ineffective conversion [69]. Since the CE compares the experimental electricity production to the theoretical electricity production from the organic compounds consumed by microorganisms in the MFC, it can be used to establish the efficiency of the microbes and feedstock. Fig. 5 shows the calculated Columbic Efficiencies (in percentage) for all four MFCs.

The results presented in Fig. 5 demonstrates that the maximum Coulombic efficiency (CE) across the four setups was 40.17 % for MFC-4, while MFC-3 exhibited the lowest CE, 14.64 %. The order of CE among the four microbial fuel cells (MFCs) is as follows: MFC-3 < MFC-1 < MFC-2 < MFC-4. This trend appears to correlate positively with the percentage of Chemical Oxygen Demand (COD) removal, suggesting that higher COD removal percentages are associated with enhanced CE values. Notably, Ishaq et al. [70] reported analogous but lower CE values of 25.5 % and 13.5 % corresponding to COD removal percentages of 89 % and 72 %, respectively, while utilizing leachate as a substrate in a Nafion-117 membrane system. The variability in CE values can be attributed to the differential amounts of COD removal and the current generated, assuming other factors remain constant. Specifically, an increase in ΔCOD tends to decrease CE, whereas an increase in current at a constant ΔCOD results in an elevated CE value. Furthermore, a statistically significant p-value (p=0.02) was noted for the CE measurements across all four MFCs, underscoring the relevance of the observed differences. In conclusion, the findings indicate that approximately 50 % of organic matter can be effectively converted into electrical energy, underscoring the potential of utilizing mixed blackwater and agricultural waste for bioelectricity production.

**Fig. 6:**
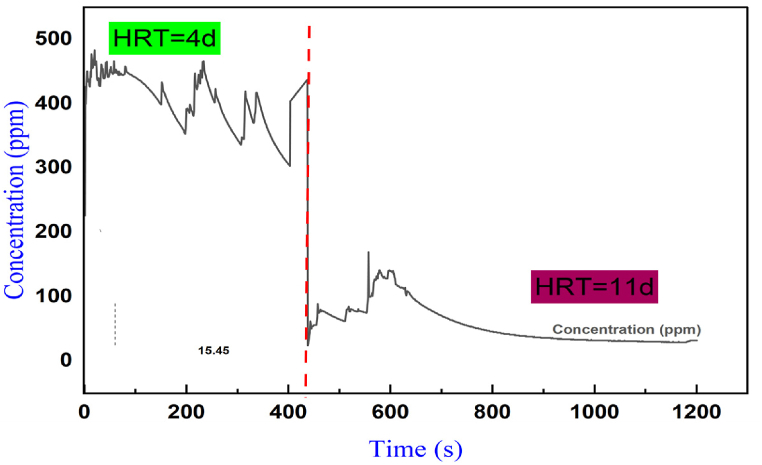
BioH_2_ produced after four days of fermentation (HRT = 4) and after 11 days of fermentation (HRT = 11) for 300 g of cassava peel, 100 g of banana, 200 g of tomatoes, and 200 ml of blackwater.

During the biocatalytic conversion of biomass to produce energy, some microorganisms can produce CH_4_, CO_2_, and electrons, while hydrogenases produce bioH_2_. It is essential to consider the concentration (in ppm) of hydrogen produced to assess the efficiency of the feedstock and bioreactor [71]. H_2_ generated in parts per millionth per second was measured using the MQ-8 H_2_ gas detector supplemented by an Arduino Leonardo board and a computer having a range of detection 50-10,000 ppm and temperature control between −10 and 50. The H_2_ gas was collected and recorded from 0 to 400s and a concentration gradient of 0–500 ppm. Fig. 6 presents the H_2_ yield over a hydraulic retention time of 4 days (left of red vertical dotted lines) and 11 days (right of red dotted lines).

Fig. 6 shows the highest peak produced in the first 250s (483 ppm/s, 226 s). However, it starts to drop at 420s, slightly increases up to 600s, and then maintains steady state. A significant point worth noting here is that the continuous dips along the curve at HRT=200to400s with the lowest concentration observed after 400s could be due to hydrogen-producing bacteria such as *Klebsiella* [72] producing more H_2_. Meanwhile, the growth of methanogens within the bioreactor rapidly shifts to an exponential stage, which can reduce the average H_2_ concentration at a higher rate by combining the H_2_ produced with CO_2_ to produce methane via Hydrogenotrophic Methanogenesis [72].

**Fig. 7:**
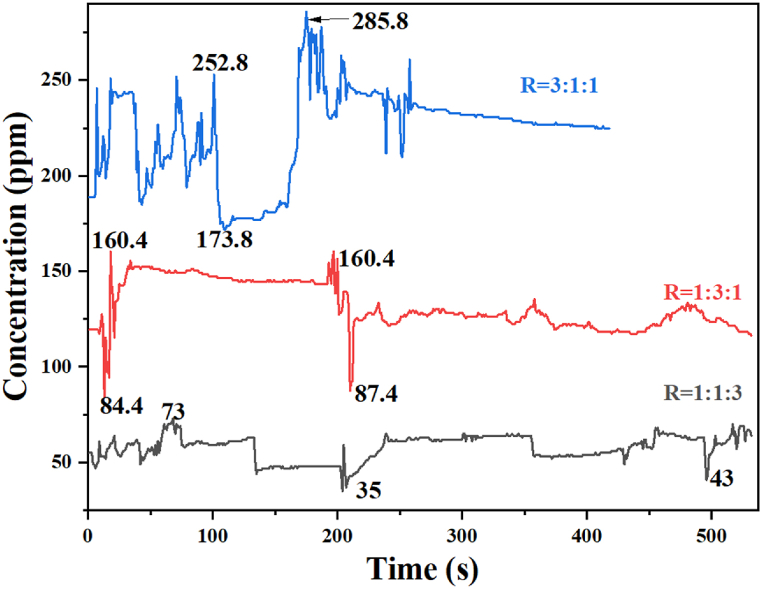
Effect of varying feedstocks ratio on H_2_ yield. Ratio (R) = Cassava peel (CP): Banana peel (BP): tomato wastes (TW).

The nutritional composition in each feedstock has been extensively reviewed in the literature. Ripe bananas have been reported to consist primarily of carbohydrates with more than 16 % sugar content [73]. Cassava, on the other hand, is a rich source of carbohydrates ranging from 86.28% to 93.13 % [74] despite its low sugar content of 1.8 g per 0.5 cups. Tomatoes (*Solanum lycopersicum* L.) are more fibrous than both banana and cassava and have been reported to contain sugars in the range 1.50–5.65 and 2.20–2.70 % for both domesticated and wide tomatoes with ascorbic acid (12.40–35.56 and 23.62–28.14 mg/100 g) [75]. The total sugars and reducing sugars have also been reported to be in the range of 30.03–41.21 % and 47.00–56.45 %, respectively. Fig. 7 shows how combining these agricultural wastes in different ratios can affect the biohydrogen yield.

Fig. 7 shows the concentration and yield of H_2_ in parts per million (ppm) per second for three different setups with varying ratios of cassava peels (CP), banana peels (BP), and tomato wastes (TW). When the mass of cassava peels was increased to 300 g while maintaining constant masses (100 g) for banana peels and tomato wastes, the H_2_ production peaked at 285.8 ppm/s, which is thrice the rate for one-third the mass of cassava in R = 1:1:3. That is, cassava peels produce thrice as much biohydrogen compared to tomato wastes *ceteris paribus*. Technically, the sample size is inadequate to jump into such a generalization; however, the findings provide the basics for comparing the hydrogen-producing potential of cassava peel versus tomato wastes versus banana peels. The second highest H_2_ production occurred when the mass of banana peels increased to 300 g, with constant masses of cassava peels and tomato wastes. These results suggest that the high H_2_ production from the 300 g cassava peel setup is due to the high concentration of carbohydrate content found in cassava compared to banana peels and tomato wastes, respectively, under the same environmental conditions [76]. Additionally, reports show that the continuous addition of sucrose can increase the average H_2_ produced in a bioreactor catalyzed by *Klebsiella oxytoca HP1*.

*E. coli* present in the community has been found to increase the growth and subsequent production of H_2_ [77]. A plateau-like trend for a ratio of 1:3:1 from 45 to 200 s and beyond may be attributed to the production of volatile fatty acids, potentially impeding the survival and growth of hydrogen-producing microorganisms, consequently reducing H_2_ production. As the concentration of sugar reduces in a ratio of 1:1:3, the overall H_2_ production also decreases. The quantity of H_2_ produced increases with the rising concentration of carbohydrates and other simple sugars, consistent with existing literature [78]. Statistically, the p-value (p=0.0007) reflects a highly significant difference in the results, which justifies the need for optimization. This finding aligns with the bioelectricity study where higher voltage and power density were observed for MFC-4, which contained a higher mass ratio of cassava peels than banana peels or tomato wastes.

**Fig. 8:**
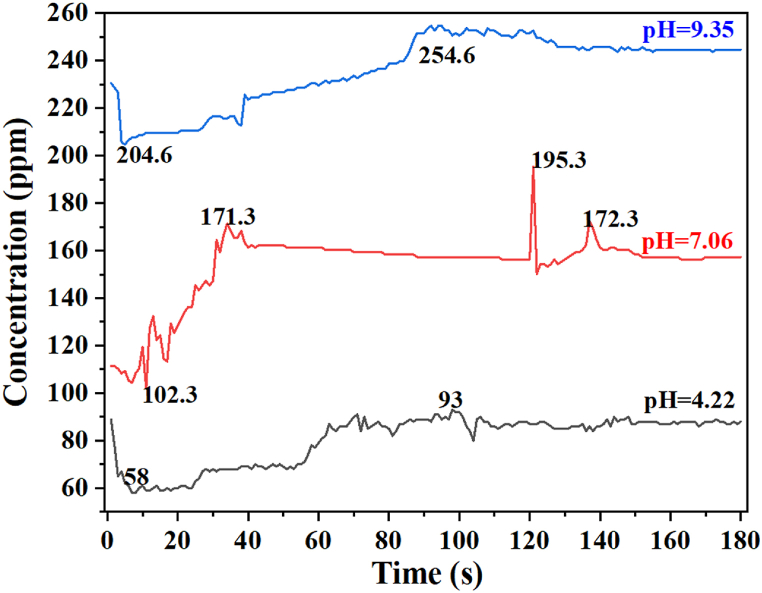
Effect of pH on H_2_ production from blackwater/agricultural wastes.

Fermentation is a biological process that involves microbial activities that are sensitive to even slight changes in pH. The pH within the reactor can affect the yield of power or H_2_. Studies have shown that lower pH values, between 6 and 9, result in higher H_2_ production, whereas pH values between 8 and 10 has been reported to increase H_2_ yield [79]. The optimal pH condition can vary for different types of wastes, with a range of 5–6 for some agricultural wastes and 7–9 for others [79]. The controversy regarding pH and H_2_ yield may be due to a lack of continuous pH control during the fermentation process. In this study, a three-point pH control system was carefully chosen, consistently monitored, and adjusted as needed. The effect of pH on the rate of hydrogen production is presented in Fig. 8.

Fig. 8 illustrates the production of H_2_ gas measured by the sensor under varying pH conditions from t = 0 to t = 180 s. The highest yield of H_2_ occurs between 100 and 140 s, indicating favorable microbial growth for H_2_ production. However, at higher hydraulic retention times (HRT), the trend in H_2_ production stabilizes with a slight decrease compared to previous values. Among the three pH conditions studied, a slightly basic pH of 9.35 resulted in the maximum H_2_ yield. This is likely due to the adaptability of Hydrogen-producing bacteria (HPB) such as *E. coli* [44] and *Enterobacter asburiae* [58] to a broad pH range. Microbial characterization results indicate that these bacteria are the sole biocatalysts found in the blackwater. Overall, a pH of 4.22 leads to the lowest H_2_ yield, as HPB struggles to survive in environments with a pH below 5 [80]. Interestingly, a higher H_2_ yield was observed in reactors with a slightly basic initial pH condition, likely due to the presence of pH-resistant *E. coli, shigella*, and *Enterobacter asburiae*. Indeed, it is apparent that optimal pH is crucial in increasing the rate of biohydrogen production and bioelectricity of wastewater, where a basic pH increases the power density of an MFC. This reasoning is statistically grounded by the low p-value (p=0.02).

## 4 Conclusion

In this study, it has been established that blackwater contains adequate microbial consortia capable of catalyzing bioelectricity and biohydrogen production, and when added to agricultural wastes already rich in nutrients, can be promising for clean energy production. An integrated dual-chamber MFC designed to accommodate the co-production of bioelectricity and biohydrogen yielded a high electricity production and high biohydrogen production rate with reasonable statistical variation (p<0.05). Microbial characterization shows the major microorganisms present included *Escherichia Coli, Salmonella spp., Pseudomonas aerogunisa, Shigella sp., Klebsiella pneumonia, Enterobacter asburiae, candida guillermondii,* and *Pythium ultimum*. These microbes have been reported to show a significant level of biocatalytic propensity during bioelectricity and biohydrogen production. The highest %COD and %BOD removals were 90.87 % and 76.67 %, respectively. The maximum open circuit voltage obtained was 1090 mV, with a power density of 345 mW/m^2^, and the highest columbic efficiency of 40.17 %. The highest biohydrogen production rate recorded was 483 ppm/s within the first 226 s of measurement. The optimum feedstock ratio was cassava Peel (300 g): banana peel (100 g): Tomato Waste (100 g) or 3:1:1. In all cases, except the %COD removal, the p-value has been far less (p≪0.05), which justify the relevance of the optimization of pH and nutrient or feedstock ratio. To the best of our knowledge, this is the first account of treating both solid and liquid wastes using a dual-chamber microbial fuel cell. This work provides a new opportunity for the application of environmental engineering to both solid and liquid waste treatment with easy-to-use technology, which is environmentally friendly and offers triple benefits: bioelectricity production, biohydrogen production, and waste treatment without any external energy.

## CRediT authorship contribution statement

**G. Plason Z. Plakar:** Writing – original draft, Methodology, Investigation, Formal analysis, Data curation, Conceptualization. **Abdulsalami S. Kovo:** Supervision, Resources. **Kanayo L. Oguzie:** Project administration. **Emeka E. Oguzie:** Writing – review & editing, Validation, Supervision, Software, Funding acquisition, Conceptualization.

## Declarations

There were no attempts made to obtain Ethics approval and consent to participate in this study, as the work did not involve humans, animals or whatsoever participants.

## Consent for publication

A Consent for Publication was not required for this study, as there were no participants involved in the study.

## Data availability

All data are available in the supporting information.

## Declaration of competing interest

The authors declare that they have no known competing financial interests or personal relationships that could have appeared to influence the work reported in this paper.
