# Supplementary material for: Valorization of mixed blackwater/agricultural wastes for bioelectricity and biohydrogen production: A microbial treatment pathway
Source: Heliyon. 2024 Dec 12;11(1):e41126. doi: 10.1016/j.heliyon.2024.e41126 (PMC11714408; doi:10.1016/j.heliyon.2024.e41126)
Supplement: Multimedia component 1 [file mmc1.docx]

**Supporting Information**

**Valorization of Mixed Blackwater/Agricultural Wastes for Bioelectricity and Biohydrogen Production: A Microbial Treatment Pathway**

G. Plason Z. Plakar^a,c*^, Abdulsalami S. Kovo^a,d^, Kanayo L. Oguzie^a,b^, Emeka E. Oguzie^a,b^*

[^a^](https://acefuels-futo.org/) African Centre of Excellence in Future Energies and Electrochemical Systems (ACE-FUELS), Federal University of Technology, Owerri, PMB 1526, Imo State, Nigeria.

[^b^](https://futo.edu.ng/) Federal University of Technology Owerri, Imo State, Nigeria.

[^c^](https://www.cu.edu.lr/)Department of Chemistry, Emmet A. Dennis College of Natural Sciences, Cuttington University, Gbarnga City, Republic of Liberia.

[^d^](https://futminna.edu.ng/) Federal University of Technology Mina, Minna, Nigeria.

Corresponding author’s email: [plakar.plasonz@gmail.com](mailto:plakar.plasonz@gmail.com)

**Table S1.** The ratio of Mixed BW/AW for four DC-MFC setups

| **MFC Type** | **CP** | **TW** | **BP** | **BW** |
| --- | --- | --- | --- | --- |
| MFC-1 | 100g | 100g | 100g | 100mL |
| MFC-2 | 200g | 100g | 300g | 400mL |
| MFC-3 | 200g | 200g | 200g | 200mL |
| MFC-4 | 300g | 100g | 200g | 400mL |

*CP=cassava peels, TW=tomatoes waste, BP=banana peels, and BW=blackwater*
